# CUBAN, a Case Study of Selective Binding: Structural Details of the Discrimination between Ubiquitin and NEDD8

**DOI:** 10.3390/ijms20051185

**Published:** 2019-03-08

**Authors:** Elena Santonico, Ridvan Nepravishta, Walter Mandaliti, Luisa Castagnoli, Gianni Cesareni, Maurizio Paci

**Affiliations:** 1 Department of Biology, Tor Vergata University of Rome, 00133 Rome, Italy; elena.santonico@uniroma2.it (E.S.); castagnoli@uniroma2.it (L.C.); Cesareni@uniroma2.it (G.C.); 2 School of Pharmacy, East Anglia University, Norwich NR47TJ, UK; nprrvn00@uniroma2.it; 3 Department of Chemical Science and Technologies, Tor Vergata University of Rome, 00133 Rome, Italy; w.mandaliti@alice.it; 4 Fondazione Santa Lucia Istituto di Ricovero e Cura a Carattere Scientifico (IRCCS), 00179 Rome, Italy

**Keywords:** biomolecular NMR, biomolecular recognition, NEDD8, ubiquitin, protein–protein interactions

## Abstract

The newly identified CUBAN (Cullin binding domain associating with NEDD8) domain recognizes both ubiquitin and the ubiquitin-like NEDD8. Despite the high similarity between the two molecules, CUBAN shows a clear preference for NEDD8, free and conjugated to cullins. We previously characterized the domain structure, both alone and in complex with NEDD8. The results here reported are addressed to investigate the determinants that drive the selective binding of CUBAN towards NEDD8 and ubiquitin. The ^15^N HSQC NMR perturbation pattern of the labeled CUBAN domain, when combined with either NEDD8 or ubiquitin, shows a clear involvement of hydrophobic residues that characterize the early stages of these interactions. After a slow conformational selection step, hydrophobic and then neutral and polar interactions take place, which drive the correct orientation of the CUBAN domain, leading to differences in the recognition scheme of NEDD8 and ubiquitin. As a result, a cascade of induced fit steps seems to determine the structural preference shown for NEDD8 and therefore the basis of the selectivity of the CUBAN domain. Finally, molecular dynamics analysis was performed to determine by fluctuations the internal flexibility of the CUBAN/NEDD8 complex. We consider that our results, based on a structural investigation mainly focused on the early stages of the recognition, provide a fruitful opportunity to report the different behavior of the same protein with two highly similar binding partners.

## 1. Introduction

The process of ubiquitin conjugation, referred to as ubiquitination or ubiquitylation, is a well-characterized post-translation modification (PTM) leading to the formation of an isopeptide bond between the C-terminal glycine of ubiquitin and a lysine side chain in the target protein. Once conjugated to a substrate, ubiquitin itself can be the target of further ubiquitination reactions. Indeed, seven lysine residues in ubiquitin can be modified by the sequential addition of ubiquitin moieties, thus generating a stretch of ubiquitins, the so-called ubiquitin chain, characterized by a specific linkage, depending of which lysine residue has been involved, branching out from the primary chain. In addition to driving the proteasomal degradation, which is the first biological effect that has been described as a ubiquitin-dependent process, ubiquitination can affect several aspects of protein function, such as the intracellular localization and transient protein–protein interactions with other binding partners [1,2]. Ubiquitination requires the concerted action of three distinct classes of enzymes, called E1, E2 and E3, dictating the ubiquitin code. This code represents the specific mode by which a substrate is modified, depending on the number of ubiquitylation sites, that is how many lysine residues are modified, and on the chain topology that is assembled by the enzymatic set. The ubiquitination profile will therefore dictate the fate of the modified substrate.

Despite the varying degrees of sequence similarity, all ubiquitin-like (Ubl) proteins share a similar three-dimensional structure, the ubiquitin superfold, called the β-grasp fold [3] and a similar set of enzymatic cascades (E1, E2 and E3) driving the conjugation process. The Neural Precursor Cell Expressed, Developmentally Down-Regulated 8 (NEDD8) is the one among the ubiquitin-like proteins (Ubls) sharing the highest homology with ubiquitin (~57% identity and ~76% similarity). The conjugation of NEDD8, called neddylation, requires an enzymatic cascade including the NEDD8-specific E1 activating enzyme (NAE), the E2 Ubc12 and the E3 enzyme RING-box protein 1/2 (RBX1/2). In contrast to ubiquitination, which affects virtually every protein, neddylated substrates are primarily members of the cullin (CUL) family, which are the scaffolds of the multi-subunit RING E3 of the Cullin RING Ligase (CRL) superfamily. Small but substantial differences in ubiquitin and NEDD8 primary sequences ensure a clear discrimination between substrates directed towards one or the other post-translational modification, thus preserving the specificity of the ubiquitination and neddylation processes [4,5,6].

Similar to all the PTMs, ubiquitin and NEDD8 codes need to be correctly interpreted by the proper protein–protein interaction networks. These networks are represented by the so-called “writers” and “readers”, respectively, the E1, E2 and E3 enzymes and the ubiquitin/NEDD8 binding partners, the latter expected to retain the same ability shown by the enzymatic sets to discriminate between ubiquitin and NEDD8. Nevertheless, several examples attest a promiscuous role of NEDD8 in degradative, regulatory and protein sorting/trafficking functions, which are processes typically regulated by the ubiquitin pathway [7,8,9,10,11,12,13].

We recently identified a novel binding domain that we called CUBAN (Cullins Binding Domain Associating with NEDD8) due to its ability to bind neddylated cullins [14]. Our structural characterization showed that, although the CUBAN domain also recognizes ubiquitin chains, it has evolutionary gained a modality of recognition of NEDD8 and neddylated substrates based on a dedicated binding site. This observation led us to conclude that, although it cannot be defined as a strictly “specific” NEDD8 binding domain, the CUBAN is “selective” for the ubiquitin-like protein since it has the capability of distinguishing it from its closer relative. To date, the CUBAN domain has been uniquely identified at the carboxyl-terminal end of a protein called KHNYN (KH and NYN containing protein). 

The results here reported are addressed to elucidate the basis of the selectivity in the interaction of the CUBAN domain with NEDD8, compared to ubiquitin. The structure of CUBAN domain alone and in complex with NEDD8 has already been determined by NMR chemical shift perturbation method of amide ^15^N resonances and described therein in detail [14]. During our previous analysis, we observed differences in the perturbation patterns of the CUBAN domain following the interaction with NEDD8 and ubiquitin. To better elucidate the molecular events driving these recognition mechanisms, we adopted the stepwise chemical shift perturbations of the ^15^N HSQC NMR spectrum of the labeled proteins, going from high molar ratio of CUBAN (15:1) down to the 1:1 ratio. 

Our results indicate that, after a slow conformational selection step, a complex interplay between interactions of different nature—mainly hydrophobic in the early steps and then predominantly neutral and polar—takes place, leading to differences in the recognition scheme of CUBAN domain toward either NEDD8 or ubiquitin. From the early stage of recognition, which appears to be markedly different, a cascade of induced fit steps seems to determine the binding preference shown for NEDD8. 

## 2. Results

### 2.1. Interaction of the ^15^N Labeled CUBAN Domain with NEDD8 and Ubiquitin

The molecular structure of the CUBAN domain in complex with NEDD8 was previously described, based on the ^15^N HSQC NMR at 1:1 molar ratio [14].

**Figure 1:**
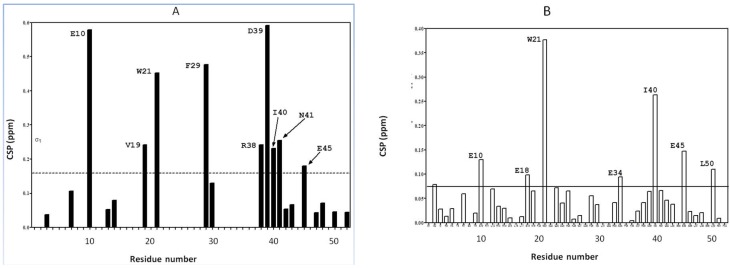
Chemical Shift Perturbations (CSPs) of ^15^N labeled CUBAN in presence of NEDD8 (**A**) and in the presence of Ubiquitin (**B**) both at a molar ratio 1:1. The horizontal line marks the one sigma of the observed CSPs. The sigma value in (**A**) (dotted) is the one used in the structure determination of the complex between CUBAN and NEDD8, as previously reported [14].

As shown in Figure 1, the inspection of the individual chemical shift perturbations (CSP) of ^15^N labeled CUBAN upon addition of NEDD8 or ubiquitin (both at 1:1 molar ratio) clearly indicates that, together with the presence of some common features, these interactions reveal significant differences. In particular, few perturbations of higher intensity characterize the CUBAN in complex with NEDD8. In contrast, numerous less pronounced perturbations, nearly diffused all along the sequence, were observed in the presence of ubiquitin. Considering the very high structural similarity between NEDD8 and ubiquitin, a common feature of all ubiquitin-like molecules, and the high sequence similarity that is actually a unique feature of NEDD8, we investigated in more detail how these differences can be interpreted in order to explain how the selectivity is achieved.

**Figure 2:**
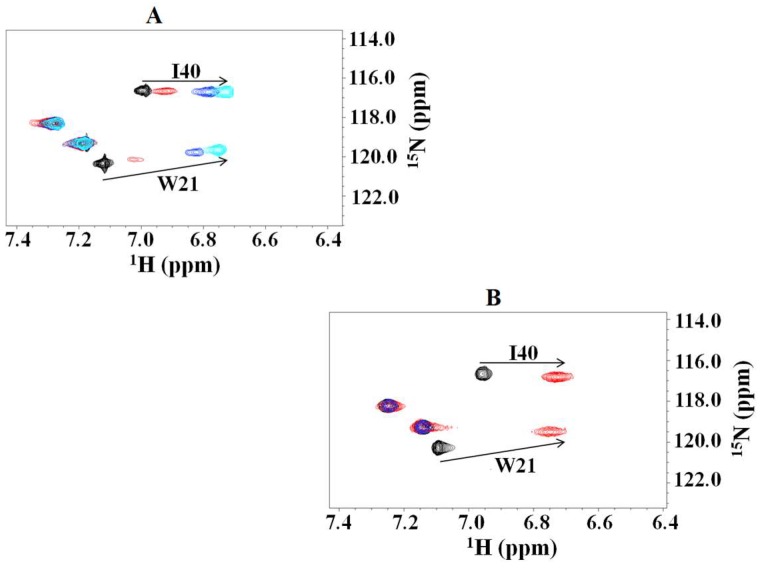
^15^N HSQC NMR of W21 and I40 in labeled CUBAN, upon stepwise addition of: NEDD8 (**A**); and ubiquitin (**B**). The molar ratios here utilized are 15:1, 8:1 and 1:1.

We first monitored the cross peaks of some single residues. As shown, W21 and I40 (Figure 2A,B, respectively) represent good examples of residues whose perturbations appear to be differently shifted, depending on the molar ratios between the two partners. Particularly, the binding profile was gradual in the complex with NEDD8, with intermediate slow exchanges, while the perturbation appeared “quickly” in the complex with Ubiquitin. On the other hand, the observation of ^15^N labeled NEDD8 upon addition of unlabeled CUBAN also reported shifts indicating a molecular interaction (Supplementary Figure S5).

Based on these preliminary results, we established a general approach based on monitoring the chemical shift perturbations (CSP) of all the resonances in ^15^N HSQC NMR spectra of CUBAN upon the stepwise addition of either unlabeled NEDD8 or ubiquitin. Our goal was to reveal the subtle differences in number and identity of hydrophobic, neutral and polar (positive/negative) residues that are involved in the early stages of the molecular recognition, which are those expected to reveal the structural determinants leading to the preference of the CUBAN domain for NEDD8. In detail, we first monitored single residues of CUBAN in the ^15^N HSQC NMR, following the addition of NEDD8 (or ubiquitin) at different molar ratios. The analysis was performed starting from a very high molar ratio of the CUBAN (15:1) until reaching the equimolar ratio 1:1. Then, the same experimental scheme was utilized for the detection of CSPs of ^15^N labeled CUBAN in the presence of ubiquitin, again at different molar ratios (from 15:1 to 1:1).

**Figure 3:**
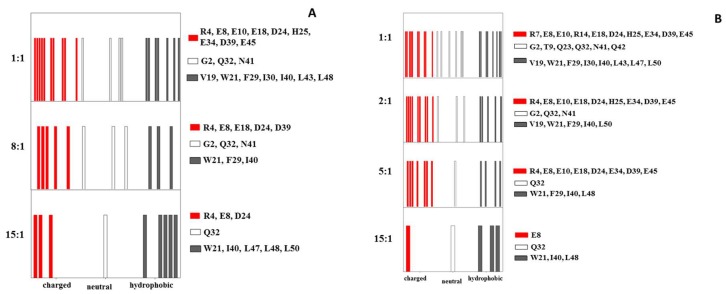
(**A**) Flags representing the residues of CUBAN domain that were perturbed in the ^15^N HSQC NMR by the interaction with NEDD8. Molar ratios from 15:1 to the final 1:1 were monitored. (**B**) Flags representing the residues of CUBAN domain perturbed in the ^15^N HSQC NMR by the interaction with Ubiquitin. Molar ratios from 15:1 to the final 1:1 were monitored. Flag colors indicate perturbed residues with polar (red), neutral (empty) and hydrophobic (black) side chains.

A similar behavior was shown by several residues in the CUBAN domain, whose spectra at 15:1 molar ratio differed depending on whether NEDD8 or ubiquitin was considered in the complex (Figures S1–S4). Results are schematically reported in Figure 3A as flags of different colors, each representing the perturbations of ^15^N resonances of the CUBAN backbone, upon addition of NEDD8 or ubiquitin (Figure 3B). The assignments of the residues corresponding to the flags are also reported. As shown, upon increasing NEDD8 concentration, an early stage mainly involving hydrophobic residues and few neutral residues was followed by the addition of several polar residues that, step by step, took part in the interaction. By comparing the flags from both experiments, we observed that the hydrophobic interactions in the early stage were more extensive upon the addition of NEDD8 compared to ubiquitin. Following the early step, an adjustment driven by several polar residues was again more populated in the case of NEDD8. Note that the comparison is to be done considering vertically the sequence of the observed effects and then making a “logical” comparison between the two experiments.

It is also important to stress the fact that the CSP method allows the detection of changes in the molecular conformation and/or in the magneto-chemical environment of the ^15^N amide atoms constituting the main peptidic chain. Consequently, only the involvement of the side chains in the molecular mechanism can be interpreted.

**Figure 4:**
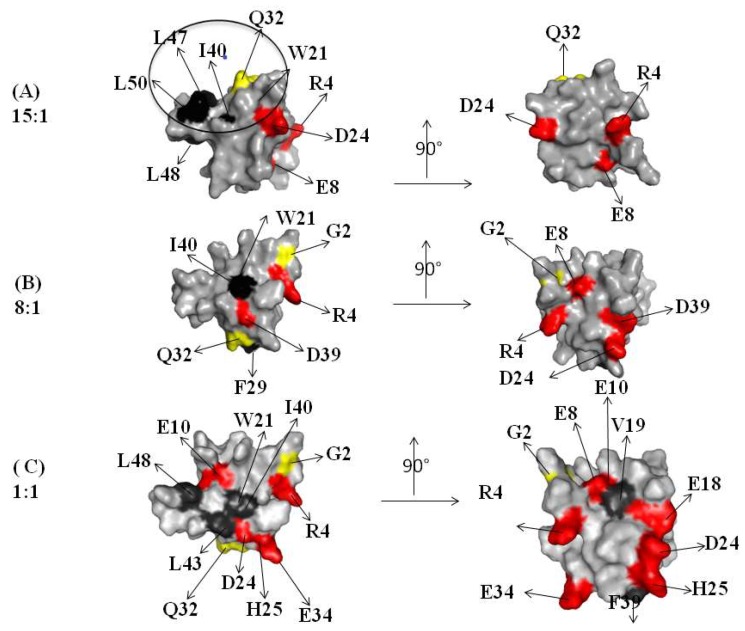
Graphical representation of the CUBAN surface regions that are perturbed in the interaction with NEDD8, upon increasing the molar ratio. The right panel shows a rotation of 90 degrees of the molecule. The color code of residues is the same as previously. The molar ratios are represented as follow: 15:1 (**A**); 8:1 (**B**); and 1:1 (**C**). A black circle indicates the binding surface involved in the interaction with NEDD8 [14].

**Figure 5:**
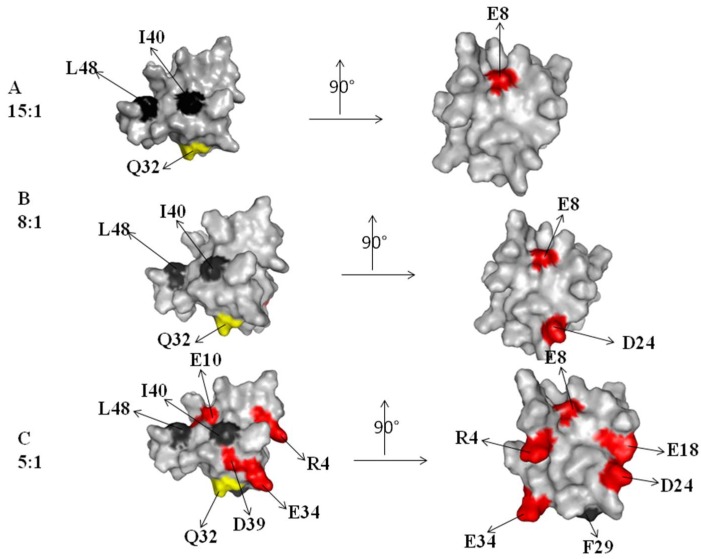
Graphical representation of the CUBAN surface regions that are perturbed in the interaction with ubiquitin, upon increasing the molar ratio. The molar ratios here reported are: 15:1 (**A**); 5:1 (**B**); and 2:1 (**C**). The right panel shows a rotation of 90 degrees of the molecule. The color code is the same as previously.

A contribution in the elucidation of the molecular mechanism arises from the molecular surface representation of the CUBAN. Figure 4 and Figure 5, respectively, show the spatial distribution, all around the protein, of the residues influenced by the interactions with NEDD8 and ubiquitin at different molar ratios (compare these residues with flags in Figure 3A,B). Figure 4 shows the regions that were perturbed in the CUBAN domain in the presence of NEDD8, together with the binding site involved in NEDD8 recognition that has been previously described (indicated by a circle) [14]. As shown, at the 15:1 molar ratio, a limited region of perturbed hydrophobic residues was visible together with a far effect involving residues D24, E8 and R4. At 8:1 mole ratio, we observed an increase in the number of perturbations, which to some extent shifted to the opposite face of the domain, accompanied by additional far effects affecting polar and neutral residues. Once the 1:1 molar ratio was reached, many other residues were perturbed all around the protein surface. For the CUBAN/ubiquitin complex, only a limited region of hydrophobic residues was perturbed (I40, L48) at the 15:1 molar ratio (Figure 5). This perturbation extended to Q32, D24 and E8 at 5:1 molar ratio, until reaching several residues all around the protein at the 1:1 molar ratio. Therefore, by focusing on similarities and differences in the interacting partners, we can clearly suggest that different binding mechanisms took place. Interestingly, some perturbed residues mapped far from the binding surface in the 1:1 CUBAN/NEDD8 complex whose molecular details has been already reported [14]. These far effects may result from perturbations of the intramolecular interaction networks (ion bridge, hydrogen bonds, and dipolar interactions) determined, both directly and indirectly, by interactions of the side chains upon binding.

### 2.2. Interaction of ^15^N Labeled NEDD8 with the CUBAN Domain

**Figure 6:**
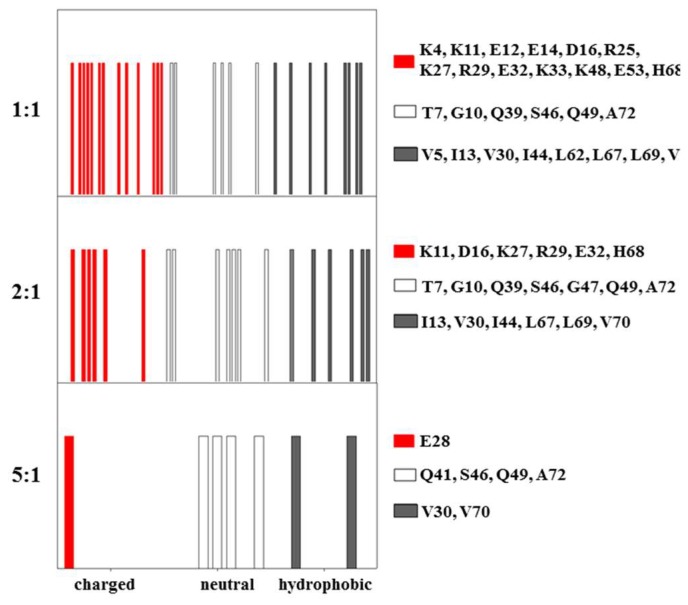
Flags reporting the residues of labeled NEDD8 that are perturbed in the ^15^N HSQC NMR upon the interaction with unlabeled CUBAN. The molar ratios CUBAN:NEDD8 are 5:1, 2:1 and 1:1, respectively. Colors indicate perturbed residues with polar (red), neutral (empty) and hydrophobic (black) side chains respectively.

**Figure 7:**
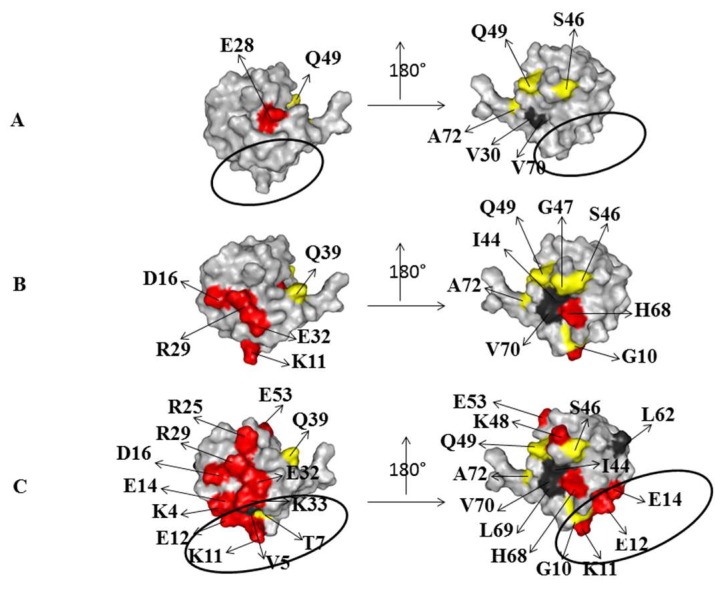
Molecular surface regions of ^15^N labeled NEDD8 that are perturbed upon the interaction with CUBAN, at the molar ratios CUBAN:NEDD8 of: 5:1 (**A**); 2:1 (**B**); and 1:1 (**C**). Color code is the same as in Figure 6. Black circles define the surface region of the interaction with NEDD8 found in the CUBAN/NEDD8 complex [14].

To complement the previous observations, the resonance perturbations of labeled ^15^N NEDD8 with unlabeled CUBAN were also monitored, using an interval of molar ratios from 5:1 to 1:1 (CUBAN:NEDD8), a range outside of which we could not detected any effect on resonances. The flags of the interaction at different molar ratios, for the CUBAN:NEDD8 molar ratios of 5:1, 2:1, and 1:1, are shown in Figure 6 (respectively, bottom, middle and top panels). The results confirm that, in the early stage of the interaction, hydrophobic and neutral residues in NEDD8 played a relevant role in the intermolecular recognition. As the molar ratio increased toward 1:1, we observed a growing number of perturbations involving several regions in the NEDD8 molecule. As shown in the molecular surface representation of NEDD8, herein reported for each of the molar ratios adopted in the case of study (Figure 7), the presence of far effects indicates that a perturbation pathway along the network of intermolecular interactions occurred, as already observed for the CUBAN domain. At the 5:1 molar ratio, only the hydrophobic residues V30 and V70 were perturbed with few detectable far effects. Unexpectedly, at this step of the interaction, we could not detect any perturbation of the residues that take part to the binding site (indicated by a circle). At the 2:1 molar ratio, several polar residues all around the protein were perturbed accompanied by several far effects on the opposite side of the molecular surface. Finally, at 1:1 molar ratio, many perturbations affected polar residues located in the binding site (V4, T7, K11, K33, E12 and E14), together with the hydrophobic residues L62 and L69, which are in proximity of the binding site.

**Figure 8:**
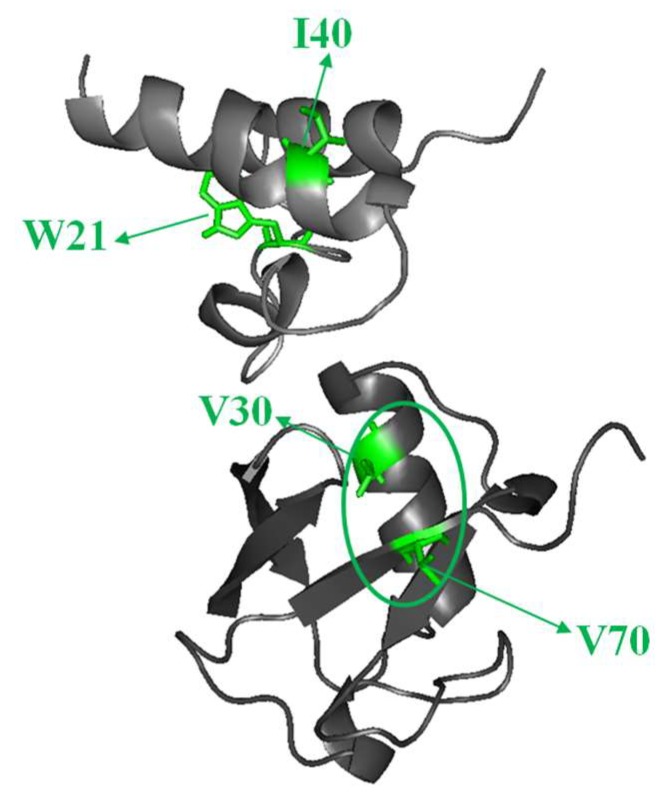
The hydrophobic residues that are perturbed in the interaction between CUBAN and NEDD8, as revealed by the ^15^N HSQC perturbation map, are mapped on the cartoon representation. Residues were selected among those perturbed in the interactions of labeled CUBAN with NEDD8 (15:1) and of labeled NEDD8 with CUBAN (1:5).

**Figure 9:**
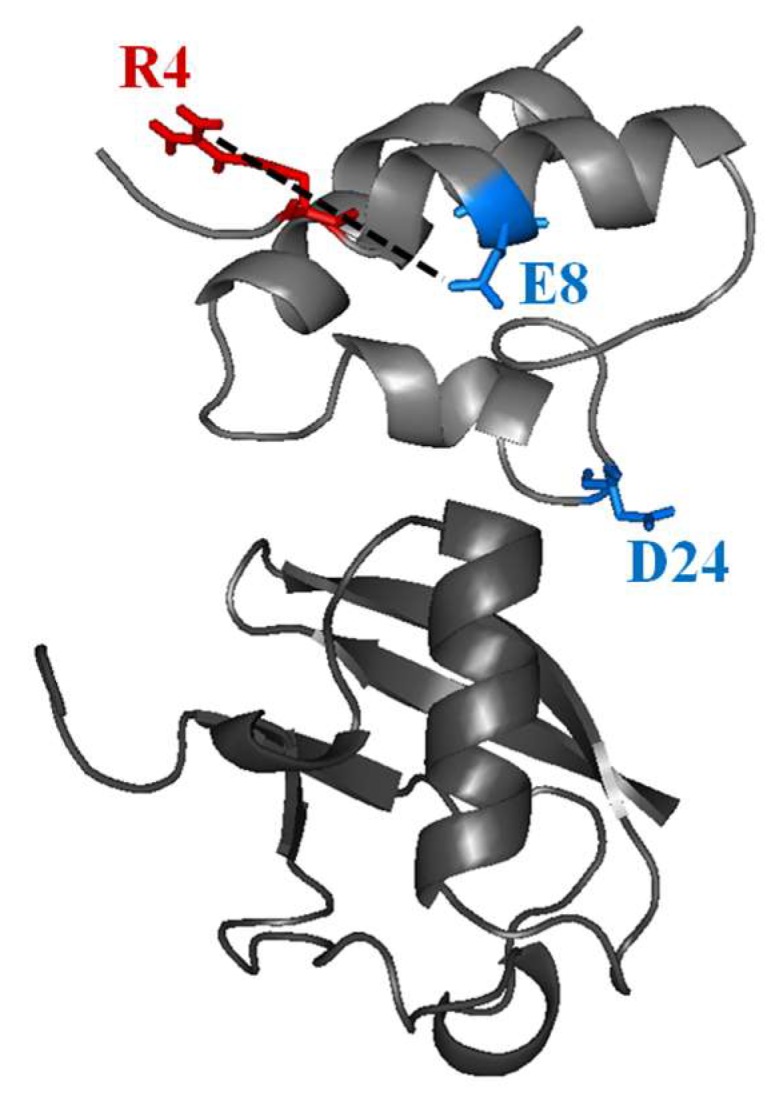
Location of the perturbed polar and neutral residues in the interaction of CUBAN domain with NEDD8. Residues R4, E8 and D24 are shown. The dotted line indicates a possible intra molecular hydrogen bond.

**Figure 10:**
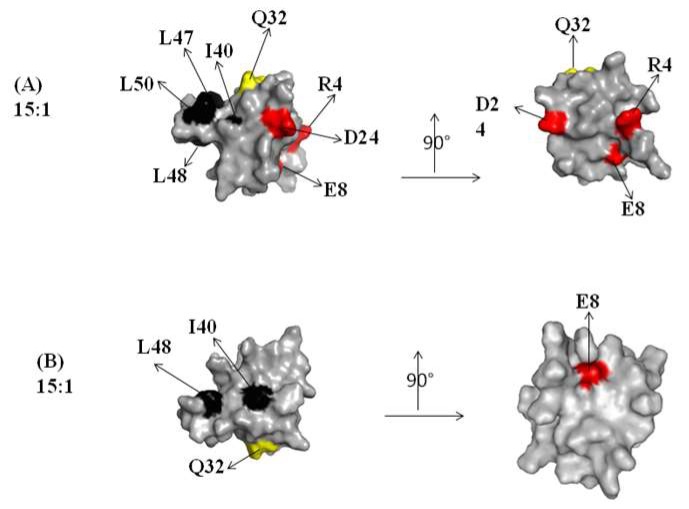
Graphical representation of the residues that are perturbed in the early stages of the CUBAN:NEDD8 (**A**) and CUBAN:Ubiquitin complexes (**B**), as revealed by the ^15^N HSQC perturbation map.

The inspection of the molecular details of CUBAN in complex with NEDD8 and ubiquitin let us depict a model of the early stage of these interactions. Figure 8 and Figure 9, respectively, report the hydrophobic and polar/neutral residues of CUBAN involved in the different stages of the interaction with NEDD8. As shown, the involvement of hydrophobic residues was clearly visible around the contact region in the CUBAN/NEDD8 complex. Moreover, the presence of an H-bond bridge between the perturbed residues R4 and E8, mapping far from the contact region, was likely to arise from the perturbation of the network. The comparison of the surface representation of the early stage interactions of CUBAN domain with either NEDD8 or ubiquitin is reported in Figure 10. The differences appeared relevant in terms of number and quality of residues involved, suggesting that the preference of the CUBAN domain for NEDD8, with respect to ubiquitin, is likely to be already determined at the early stage of the interaction.

**Figure 11:**
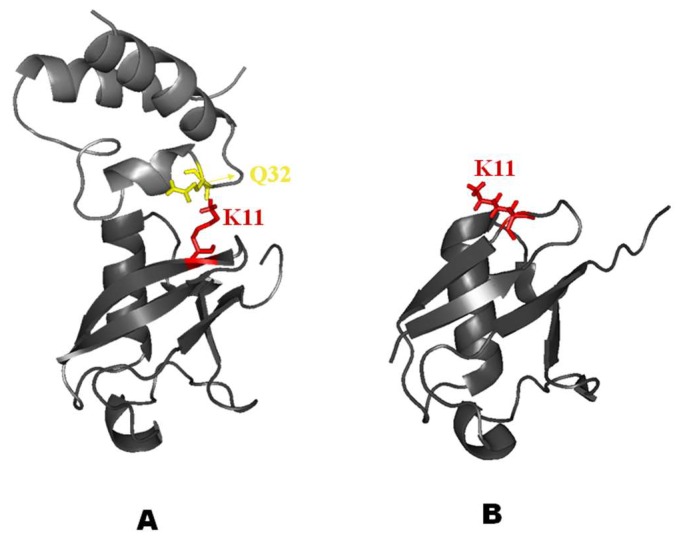
Molecular details of the interaction between CUBAN domain and NEDD8 as revealed by ^15^N HSQC NMR. (**A**) Possible intermolecular bridge due to a H-bond between the CO of Q32 of CUBAN and the NH+ of K11 of NEDD8. (**B**) Difference in local flexibility of K11 due to its the structure in ubiquitin with respect to NEDD8 (see text for details).

Finally, Figure 11 reports the possible intermolecular interaction generated between residues Q32 in CUBAN and K11 in NEDD8. This polar noncovalent H-bond interaction between the carboxy group and the positive charge of the NH_2_ (H-bond CO^…^HN+) of the side chains involved was identified by CSPs perturbations in the CUBAN/NEDD8 complex. As shown in Figure 11, while residue K11 is located in a very rigid secondary structure (β-strand) in NEDD8 (Figure 11A), the same residue maps in a flexible tract in the ubiquitin structure (loop) (Figure 11B). The different behavior of this polar bridge, which was expected to generate from a different degree of local flexibility in the formation of the two complexes, could give rise to at least some of the differences in the far effects observed in the interaction. It is important that this possible interaction should have an active role after the early stage contributing to the subsequent orientation of NEDD8 on CUBAN as well as the selectivity.

### 2.3. Structural Dynamics of the CUBAN Domain in Complex with NEDD8

**Figure 12:**
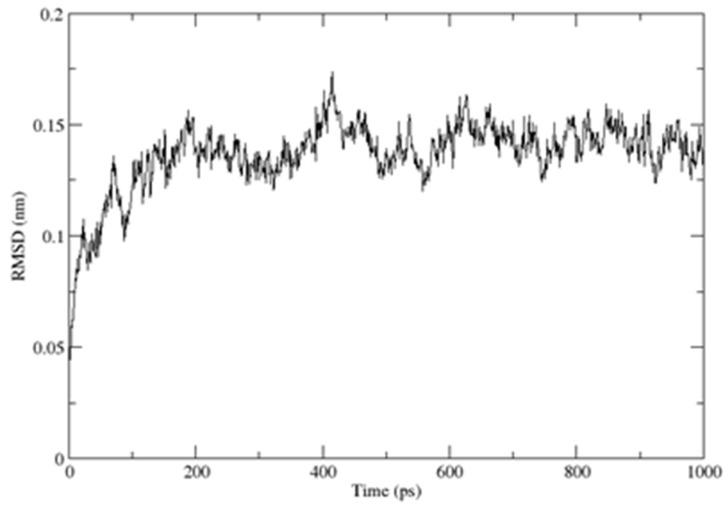
Backbone’s RMSD deviation of the CUBAN domain during the molecular dynamics simulation during time indicated in pico seconds (ps). The structure was deposited in BMRB and PDB data banks with numbers 25722 and 2N5M, respectively [14].

**Figure 13:**
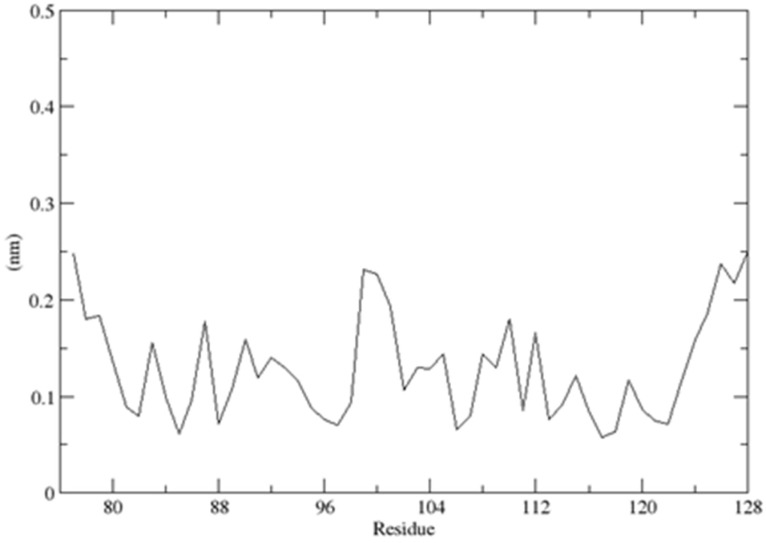
Mean fluctuation of individual CUBAN residues during the molecular dynamic simulation.

As previously described, the CUBAN domain consists of a three-helix bundle connected by two loops, reminiscent of the typical ubiquitoin binding domain (UBD) structure, with the three helices spanning residues T6–R14 (α1), K26–L31 (α2) and Y36–E52 (α3), respectively [14]. The description of the CUBAN/NEDD8 complex and the CUBAN domain alone is previously reported [14]. By using molecular dynamics simulation in water, performed with the GROMACS package for molecular dynamics at 300 K, we refined the structure in solution of CUBAN, by including information regarding the molecular stability and the internal flexibility of the protein domain. These results are reported, respectively, in Figure 12 and Figure 13. As shown, the RMSD of the backbone fluctuated during the simulation, reaching the stability around the value of 1.3 Å (Figure 12). This value remained stable during all molecular dynamics simulation, thus indicating that the system quickly reached its equilibrium at such temperature and environment. Fluctuations of individual residues of the CUBAN domain during the simulation are reported in Figure 13. As shown, in addition to the two terminal ends, we observed a marked flexibility of the four-amino acid stretch in the first loop, including residues from G22 to H25.

**Figure 14:**
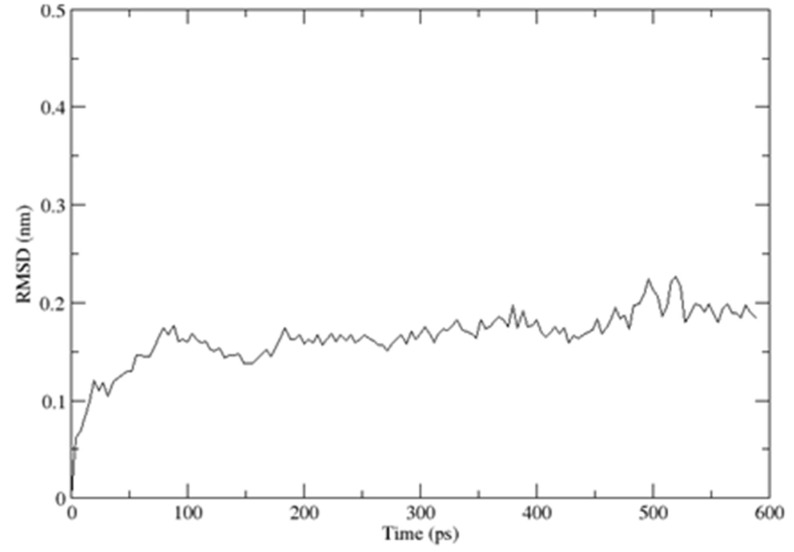
Distribution of backbone’s RMSD deviation during the molecular dynamic simulation of the CUBAN/NEDD8 complex during time indicated in pico seconds (ps).

**Figure 15:**
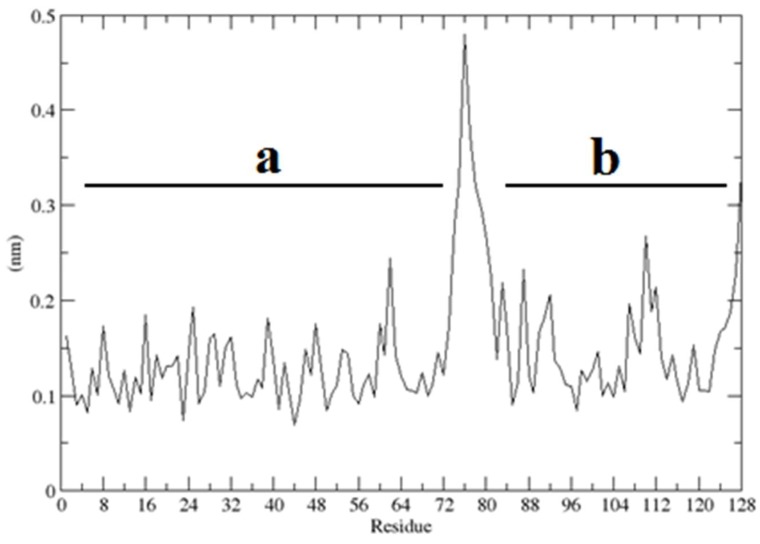
Mean RMSD fluctuations per residue of NEDD8 (**a**) and CUBAN (**b**) in the molecular dynamics simulation. Tracts with RMSD > 0.3 nm are located in the C-terminal end of NEDD8 and in the N-terminal end of CUBAN.

**Figure 16:**
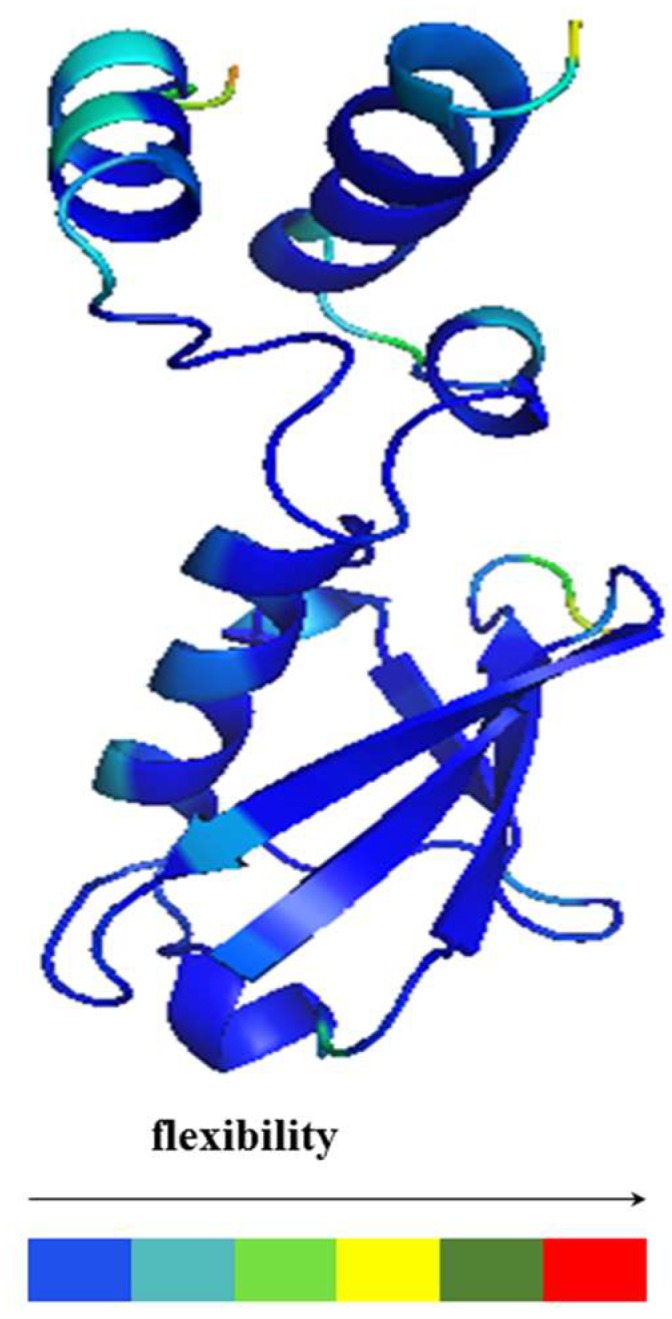
Internal flexibility of the CUBAN/NEDD8 complex obtained by molecular dynamic simulation, superimposed to the structure as obtained by NMR spectroscopy (25808 and 2n7K) [14]. The differences in the backbone flexibility of the molecular moieties are visualized by color scale.

The same procedure was used to determine the stability of the complex, where the RMSD quickly reached the stable value of 1.5 Å in such temperature and environment (Figure 14). The root mean square fluctuations (RMSF) per residue, measured in the complex of the CUBAN domain with NEDD8, are reported in Figure 15. As shown, the higher fluctuations involved the C-terminal region of NEDD8 and both terminals of CUBAN. A relatively high fluctuation was also observed in the central region of the CUBAN domain that was engaged in the interaction. These regions of flexibility, distinguished by a color code, are graphically represented in the cartoon in Figure 16, which reports the structure of the complex already published [14].

## 3. Discussion

Non-covalent interactions between proteins are the central physicochemical phenomenon underlying biological signaling. Based on the information from the study of the CUBAN domain in complex with NEDD8 and ubiquitin, we performed a structural analysis of these interactions at different steps of the recognition. From our view, the interpretation of our results leads to more general considerations in the field of protein–protein recognition. In particular, we refer here to those interactions involving flexible protein as CUBAN with binding partners that share high structural similarity, as in the case of NEDD8 and ubiquitin. This study therefore provides an opportunity to report the different behavior of the same protein with two highly similar binding partners mainly in the early stages of the recognition. The results shown here suggest that hydrophobic interactions taking place in an early stage of binding are followed by several ionic and dipolar interactions that drive the recognition pathway towards the final complex.

### Conformational Selection or Induced Fit?

The results reported here indicate that sequential steps substantiate the binding selectivity of CUBAN toward NEDD8 and ubiquitin. These steps were clearly revealed by the CSPs study obtained during the transition from a low mole ratio, the early step, to the 1:1 interaction. The early step, mainly defined by a slow exchange kinetic involving hydrophobic contacts, evolved toward a more stable structure involving a complex interaction network among hydrophobic, polar and non-polar residues that differ depending on whether NEDD8 or ubiquitin is considered. Interestingly, the number of perturbed regions suggests the involvement of several other residues that did not necessarily map around the interaction site. Moreover, as the molar ratio reached the 1:1 interaction, differences between the two cases of study were more pronounced (Figure 3, Figure 4 and Figure 5). For these reasons, we expect that the preference for NEDD8 shown by the CUBAN domain, at least when compared to monomeric ubiquitin, resides in the specific transition from the early stage to the final complex.

As already noted, the CSPs method allows the detection of changes in the molecular environment of the ^15^N atoms of the main peptidic chain, thus revealing a change of either their conformation or magneto-chemical environment. Accordingly, the perturbation propagates to all of the interaction regimes (ion bridges, hydrogen bonds, and network of dipolar interactions) and primarily involving the side chains inside the molecule. Consequently, small conformational changes of side chains at one site can be transmitted to other sites of the molecule by both internal interactions and backbone conformational adjustments. To this end, and to rationalize the perturbation pathway along the network of intermolecular interactions and ionic bridges observed in the interaction with NEDD8, we performed a detailed analysis of the residues that were perturbed in NEDD8, as found in the solution structure (pdb code 2KO3), together with a comparison of the same proximities in the crystal structure (PDB code 1NDD) (Table S1). This analysis showed that in NEDD8 (and in ubiquitin due to the high homology) the distances between the charges of the residues changed markedly due to the physical status of the protein constricted in a crystal with a reduction of many degreed of flexibility. Therefore, in the solution complex, many electrostatic bridges that are present in both proteins can be altered, thus affecting the local chemical shift perturbation that can be lastly propagated to the ^15^N of the backbone [15]. Based on these observations, we can interpret the far effect as due to a complex network of perturbations affecting strong and weak interactions present in the secondary and tertiary structure of the protein. In this context, all of the observed effects resulted from a direct or indirect perturbation induced by the interaction of the CUBAN domain with NEDD8 and vice versa.

In conclusion, a relevant number of residues of similar nature were involved in the binding of CUBAN with both NEDD8 and ubiquitin, at 1:1 mole ratio. We propose a model involving a first step characterized by a conformational selection induced by hydrophobic interactions (in the CUBAN domain but also in NEDD8 and ubiquitin), with a slow exchange dynamics in the NMR timescale, followed by a pattern of polar and neutral residues involving a network of hydrogen bonds (or dipolar interactions) affecting regions of the molecule not necessarily close to the binding site. All together, these observations confirmed the statement, based on ubiquitin motional studies, that the conformational selection is at the basis of the interactions between ubiquitin and proteins containing ubiquitin-binding domains [16,17]. More in detail, it has been reported that ubiquitin undergoes conformational changes that are significantly more pronounced when compared with the whole molecule on average, demonstrating that these induced-fit structural adjustments are comparable in magnitude to a conformational selection. Based on these assumptions, both the “induced fit” mechanism (binding first) and the “conformational selection” (conformational change first), which are the two interactions schemes proposed by Jean-Pierre Changeux and Stuart Edelstein [18], can be interactively mixed and further complicated by other independent dynamical features, thanks to the high internal flexibility/mobility of ubiquitin as found both in solution and in the solid state [17,19]. Due to the high sequential similarity, the same concept can be applied to NEDD8.

Interestingly, a wide range of conditions has been identified in the cases of small rigid molecules interacting with proteins. In those cases, the mechanism switches from being dominated by the conformational selection pathway, at low ligand concentration, to induced fit at high ligand concentration [20].

Moreover, due to the internal motions of ubiquitin, a more complex mechanism was further hypothesized that again might be similarly extended to NEDD8 [21]. In fact, ubiquitin in complex with 11 different binding partners was compared with ensembles of unbound ubiquitin by molecular dynamics simulations on a microsecond timescale. As described by the authors, along the main mode of fluctuation, in most cases, ubiquitin binding reduces its available conformational space to that covered by the unbound form, thus indicating a conformational selection. Nevertheless, the differences between bound and unbound structures exclusively map at the binding interface.

## 4. Materials and Methods

### 4.1. Expression Plasmids and Protein Purification

Cloning and expression of the amino acid region spanning residues 627–678 of KHNYN was performed as previously described [14]. The recombinant protein was expressed in BL21 bacterial cells according to the protocol for NMR sample preparation described by Weber and collaborators [22].

### 4.2. NMR Spectroscopy

The experimental conditions were the same as previously reported [14]. ^1^H–^15^N HSQC NMR spectra of the CUBAN domain alone and in the presence of NEDD8 and Ubiquitin at different molar ratios were run at 298 K on a Bruker Avance instrument (Bruker Italy, Milano, Italy) operating at 700.132 MHz [23]. The implement of this pulse sequence with a gradient water suppression was applied [24]. To monitor the changes in ^15^N labeled amide groups in the different conditions, TOPSPIN 3.1 (Bruker Italy, Milano, Italy) and NMRView software packages (Free access software, NIH, Bethesda, Rockville, MD, USA) were used for data processing. Data analysis was performed by the guidelines described in [25,26].

### 4.3. Molecular Dynamics Simulations

All of the molecular dynamic simulations were performed by GROMACS package (Gromacs 4.5) [27] primarily designed for biochemical molecules. The program uses the AMBER03 protein, nucleic AMBER94 force field and the TIP3P model for water molecules. The simulations were performed on a total time of 5 ns. The cutoff radii were set at 0.8 nm for electrostatic interactions and 0.8 nm for Lennard–Jones interactions. Long-range electrostatic interactions were treated using the particle-mesh Ewald (PME) method. Temperature coupling was performed with a Nose–Hoover thermostat and pressure coupling was carried out with the Parrinello–Rahman barostat. The calculations were performed on a Linux PC presenting an AMD Athlon(tm) II X2 260 Processor × 2 at a rate of about 19.6 ns day^−1^.

## Supplementary Materials

The following are available online at https://www.mdpi.com/1422-0067/20/5/1185/s1, Figures S1–S4: Details of the resonances in the ^15^N HSQC NMR spectra of CUBAN. The lowest and highest molar ratios were measured. Figure S5: Details of the resonances in the ^15^N HSQC spectra of NEDD8. The lowest and highest molar ratios were measured. Table S1: Summary of the most important ionic distances between different polar residues in the PDB structures of NEDD8 and ubiquitin able to give hydrogen bonds or ionic bridges.
