# Supplementary material for: CUBAN, a Case Study of Selective Binding: Structural Details of the Discrimination between Ubiquitin and NEDD8
Source: Int J Mol Sci. 2019 Mar 8;20(5):1185. doi: 10.3390/ijms20051185 (PMC6429362; doi:10.3390/ijms20051185)
Supplement: Supplementary file 1 [file ijms-20-01185-s001.zip › ijms-438442-supplementary-final/ijms-438442-supplementary-proofed.doc]

Table S1. Summary of the most important ionic distances between different polar residues in the PdB strucures of NEDD8 and Ubiquitin.

| **Residue** | **Is in close proximity of** | **Distance A° (in crystal)** | **Located** | **Partner located** |
| --- | --- | --- | --- | --- |
| NEDD8 (1NDD) |  |  |  |  |
| K11 | E12 | 10.48 (8.9) | K11 flexible tract | E12 initial tract of strand |
| K27 | E53 | 11.11 |  | E53 flexible tract |
| K33 | E32 or E12 and E14 | 9.93, 8.27, 8.90 |  |  |
| R29 | E32 and D16 | 5.06, 8.14 (6.5) |  |  |
| Ubiquitin (1D3Z) |  |  |  |  |
| K11 | E34 | 6.15 |  |  |
| K6 | E64 | 11.23 | Initial tract of β strand | E64 Flexible tract |
| K27 | D52 | 4.86 |  |  |
| K27 | E51 | 9.26 |  | E51 flexible tract |
| K33 | D32 | 7.68 |  |  |
| K33 | E16 | 8.61 | Initial tract of β strand |  |
| K29 | D21 | 5.60 |  |  |
| K29 | E16 or E18 | 6.7, 8.69 |  | E18 Flexible tract |
